# Supplementary figures and images for: Glial Nrf2 signaling mediates the neuroprotection exerted by Gastrodia elata Blume in Lrrk2-G2019S Parkinson’s disease
Source: eLife. 2021 Nov 15;10:e73753. doi: 10.7554/eLife.73753 (PMC8660019; doi:10.7554/eLife.73753)

Fig. 3A

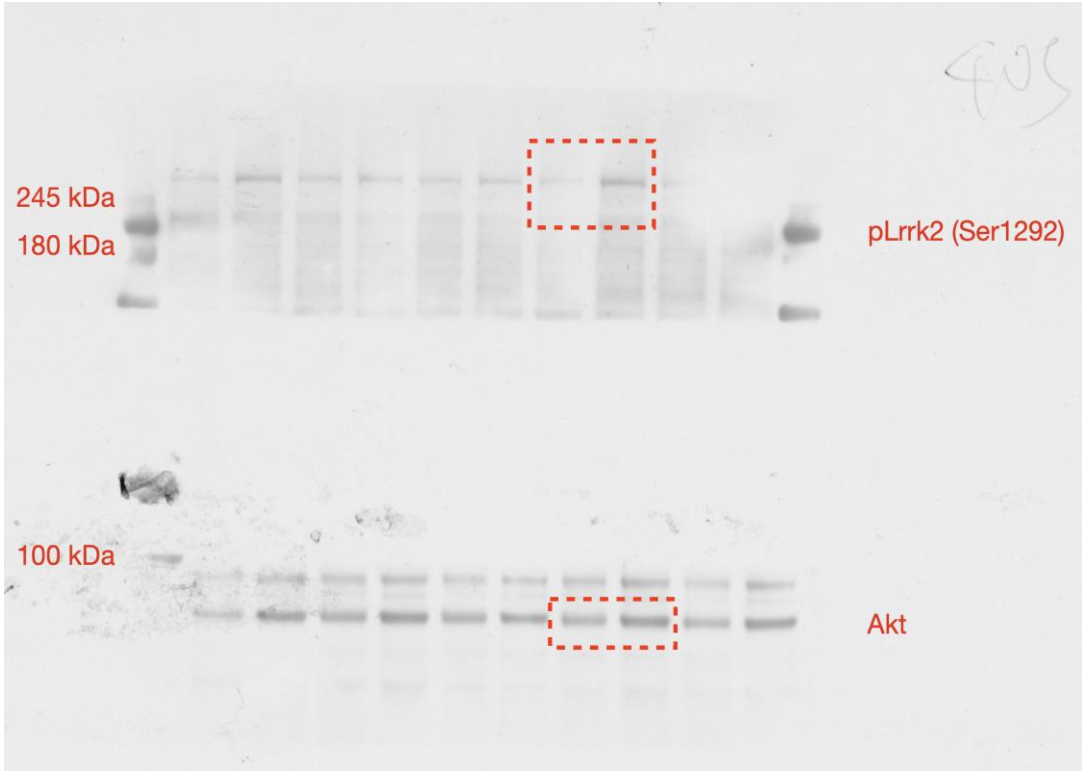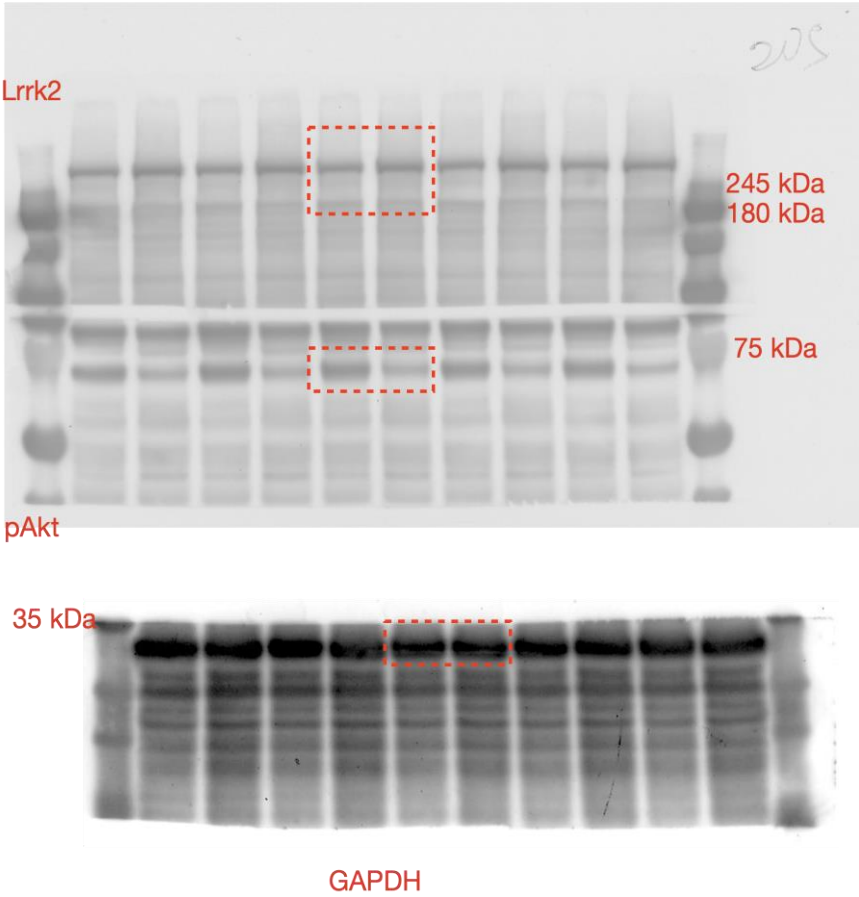

Fig. 3D

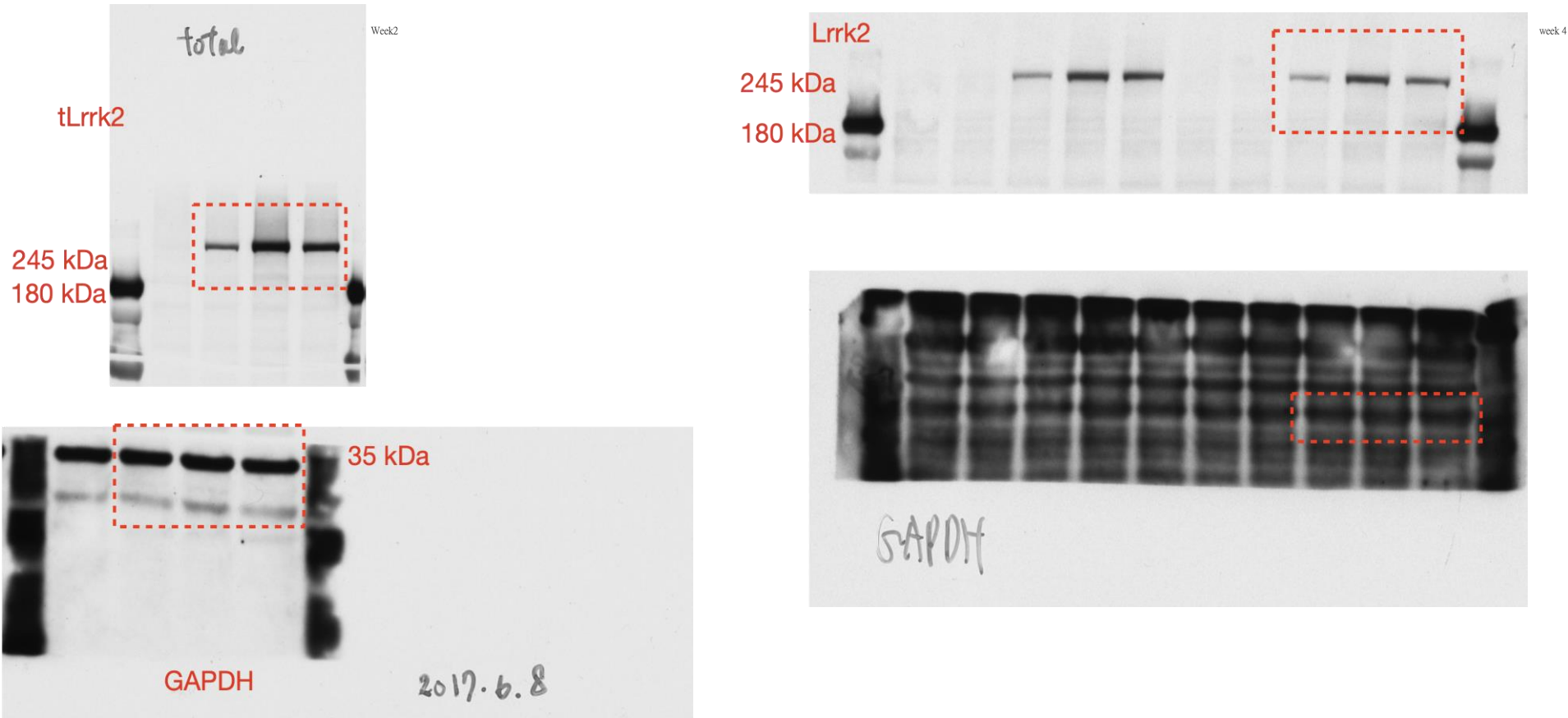

Fig. 3F

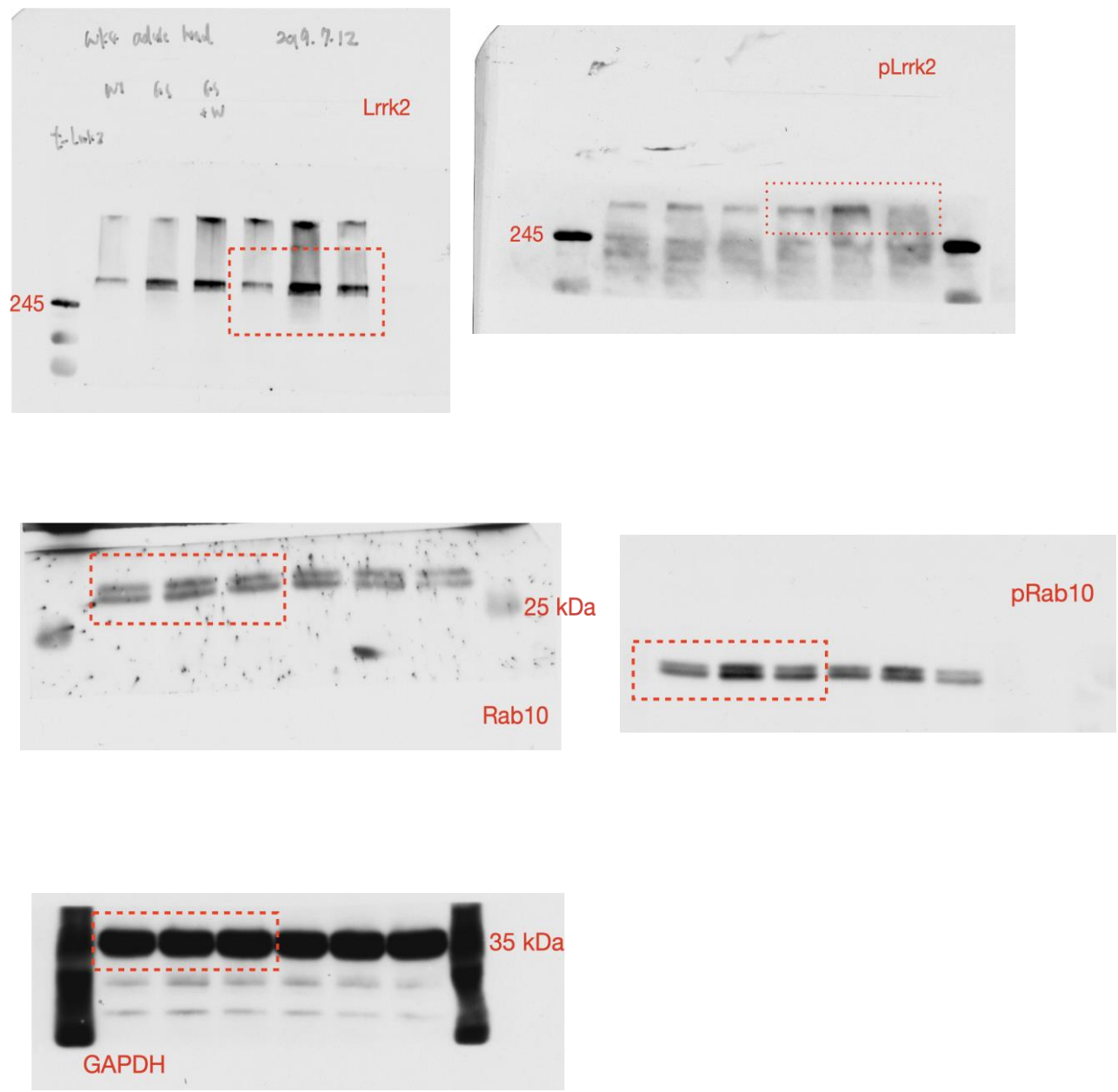

Fig. 4A

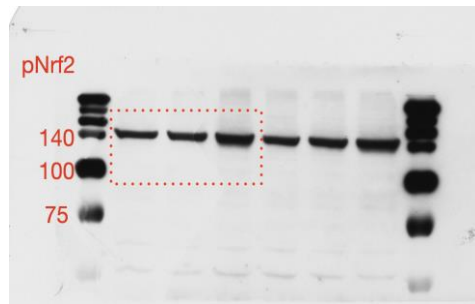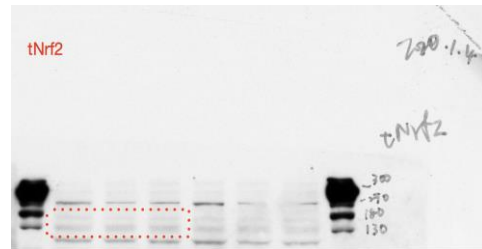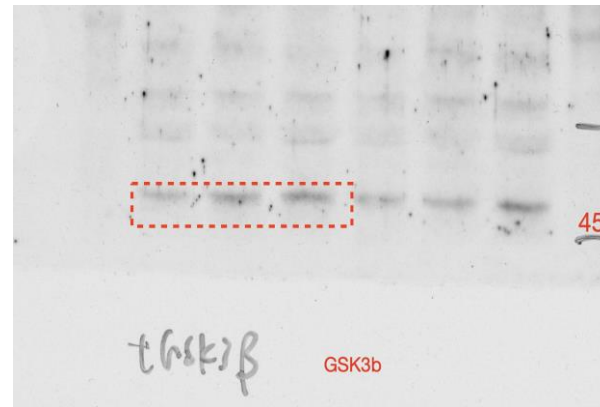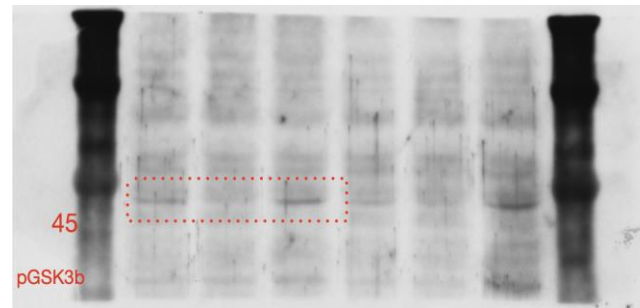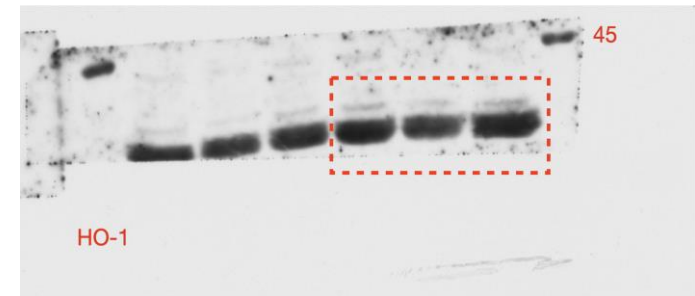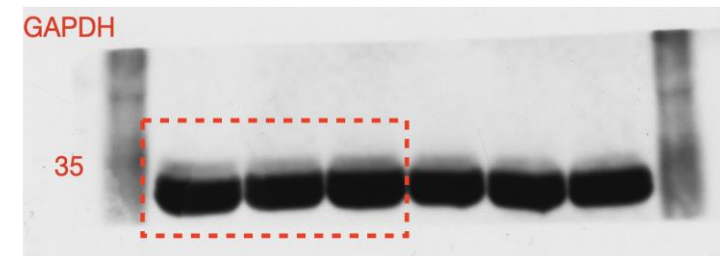

Fig. 4C

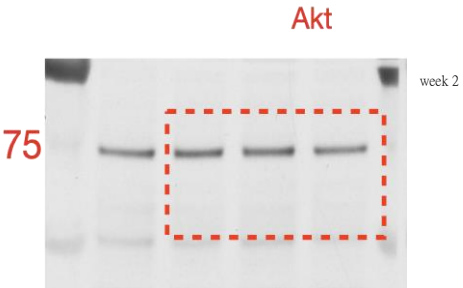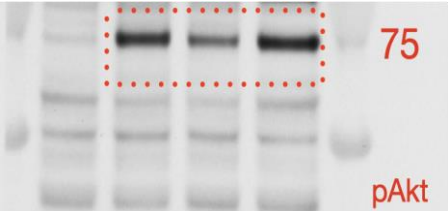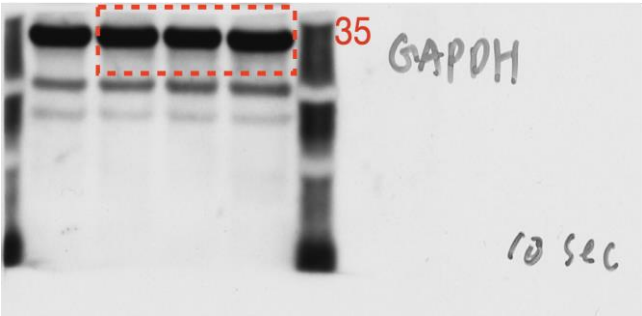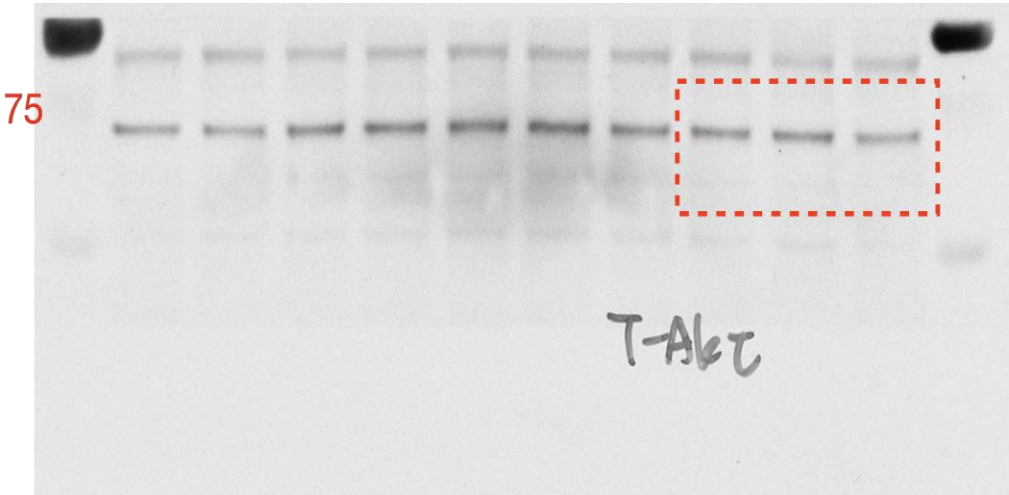

week 4

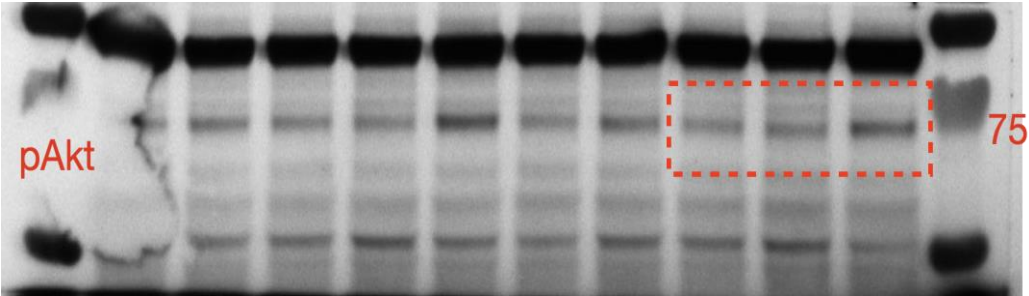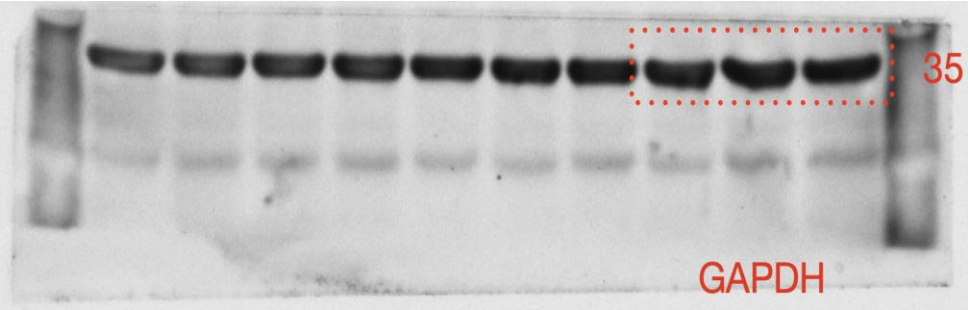

Fig. 10E

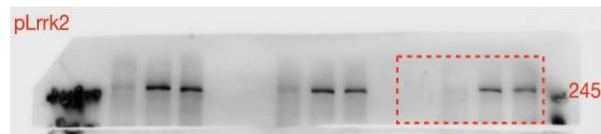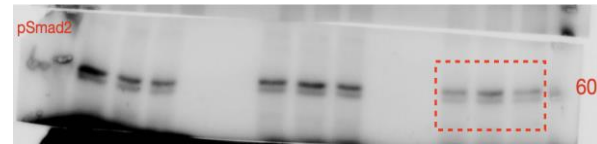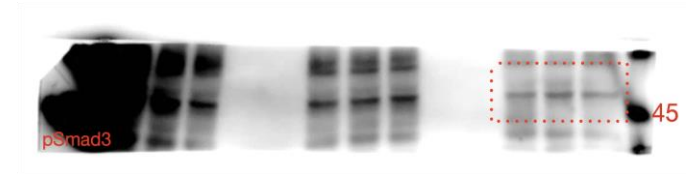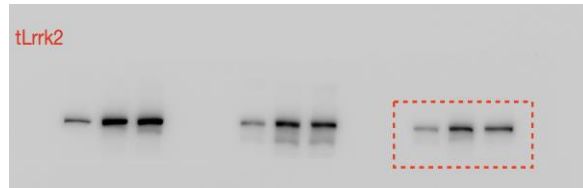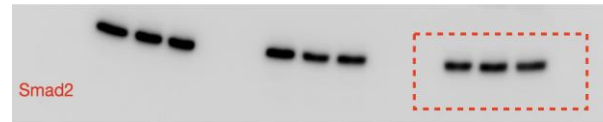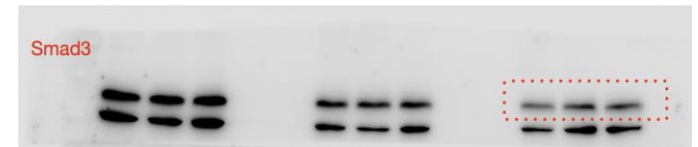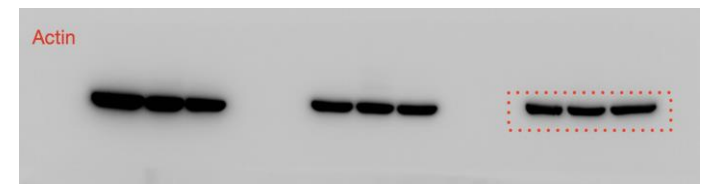

Figure 3-figure supplement 1A

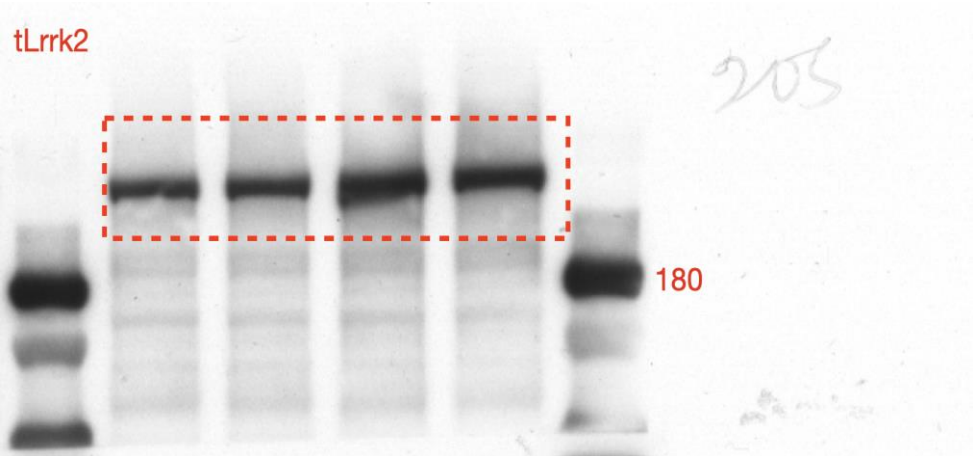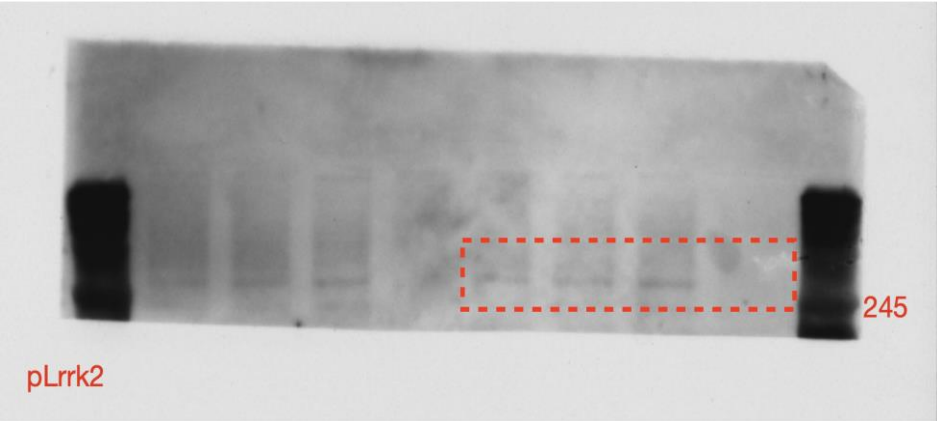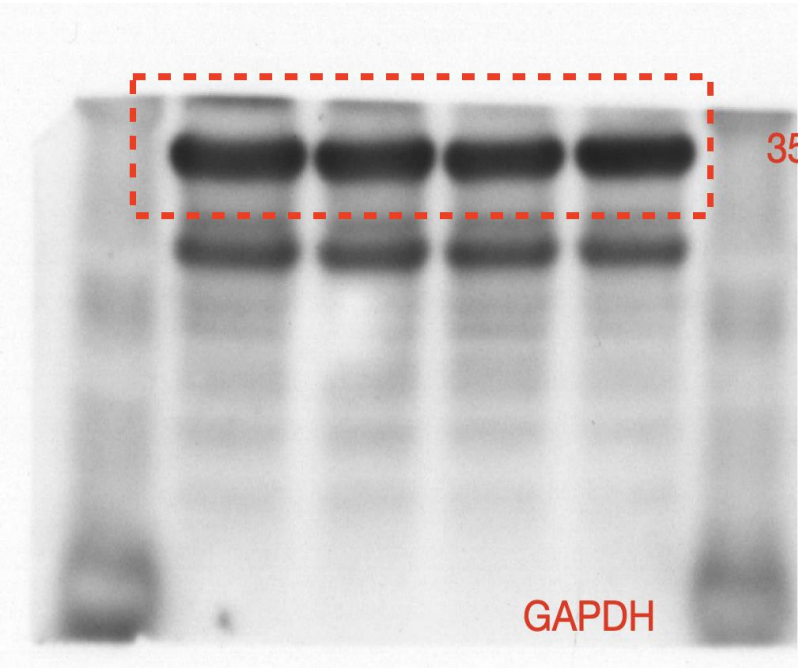

Figure 6-figure supplement 1A

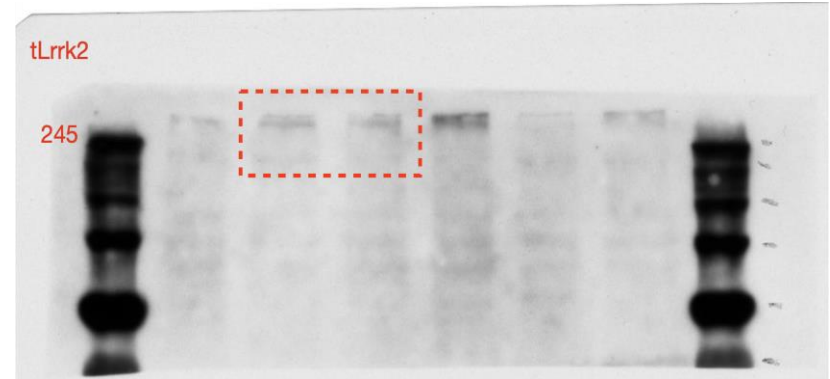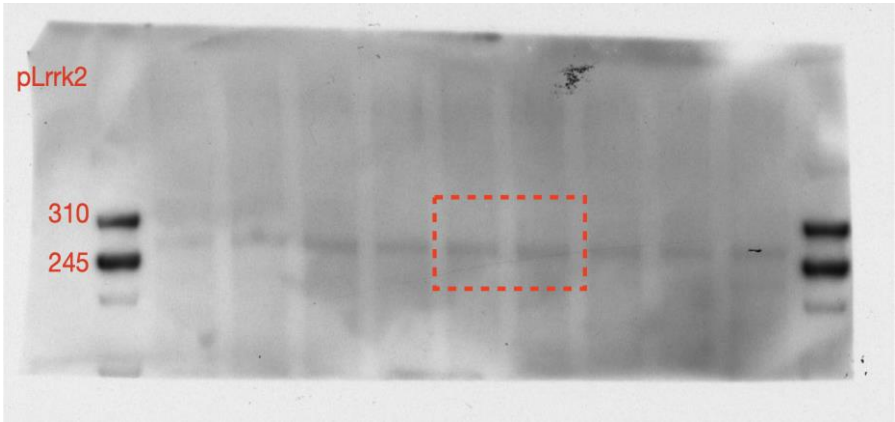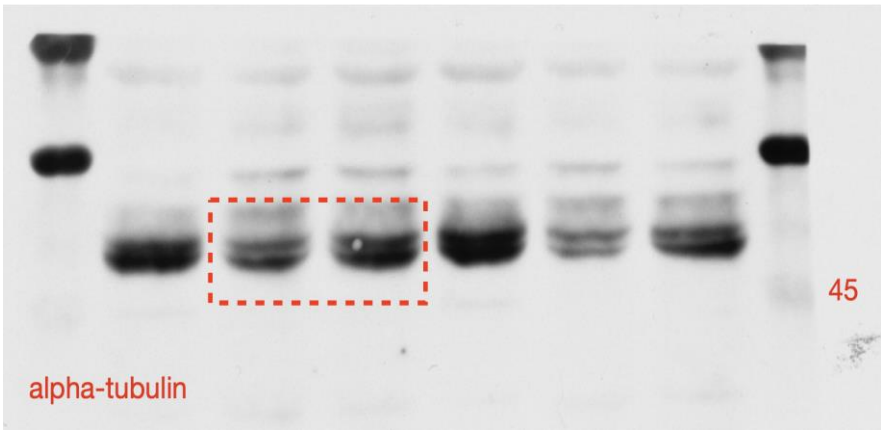

Supplement: Source data 2. — The file includes the uncropped images of the western blotting in this article. [file elife-73753-supp2.pdf]
